# Melanoma With Cardiac Metastasis

**DOI:** 10.7759/cureus.46230

**Published:** 2023-09-29

**Authors:** Sruthi L Kotaru, Jose Ricardo Po, Vikas Tatineni, Hemasri Tokala, Jagadeesh K Kalavakunta

**Affiliations:** 1 Biology, Portage Central High School, Portage, USA; 2 Cardiology, Ascension Borgess Hospital, Kalamazoo, USA; 3 Science, University of Michigan, Ann Arbor, USA; 4 Hematology/Oncology, Ascension Borgess Hospital, Kalamazoo, USA

**Keywords:** gene mutations, immunotherapy, cardiac imaging, cardiac metastases, melanoma

## Abstract

Melanoma is considered a masquerader of many diseases owing to its potential to metastasize to many organs. Several malignancies can metastasize to the heart including malignant melanoma. Historically, antemortem diagnosis of cardiac involvement of melanoma is not common, but with significant improvement in imaging modalities, the diagnosis can now be made early and accurately, aiding in treatment and improved survival. We present a case of a 36-year-old man with brief neurological symptoms and subsequent diagnosis of cerebrovascular accident (CVA). Cardiac imaging revealed incidental findings of right and left ventricular masses and lymph node biopsy, confirming metastatic melanoma. Cardioembolic etiology was suspected for his CVA. Prompt immunotherapy was initiated with improvement in his clinical condition.

## Introduction

Melanoma is a very aggressive tumor with the potential to metastasize to multiple organs. During the pre-immunotherapy era, postmortem studies showed that cardiac metastases are very common and are found in about two-thirds of the cases [1]. Unfortunately, cardiac metastases are very rarely diagnosed antemortem as the clinical presentation can be nonspecific and misleading. Prompt multimodality imaging and biopsy are essential for a proper diagnosis. Regarding treatment, depending on the tumor location and extent of invasion, chemo/immunotherapy with or without surgical intervention can be considered. We report a case of a young patient presenting with stroke-like symptoms and subsequent diagnosis of melanoma with extensive cardiac metastases, treated with immunotherapy.

## Case presentation

**Figure 1:**
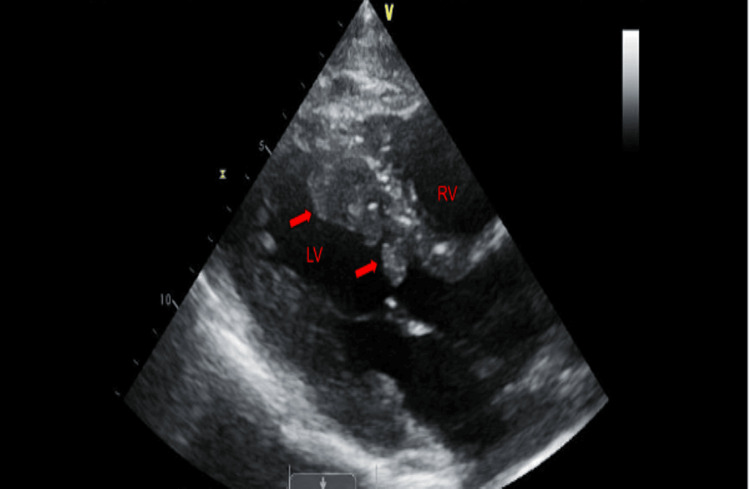
2D echocardiogram, parasternal view showing multiple masses in the left ventricular (LV) cavity. RV, Right ventricle

**Figure 2:**
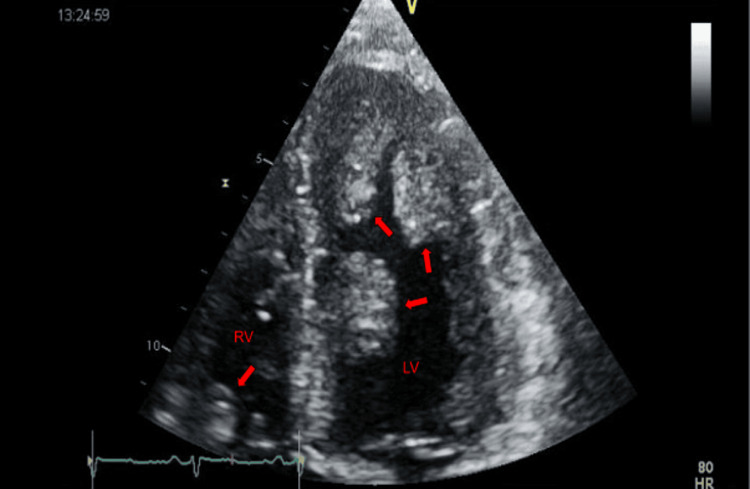
2D echocardiogram, apical four-chamber view with masses in the left ventricular (LV) and right ventricular (RV) cavities.

**Figure 3:**
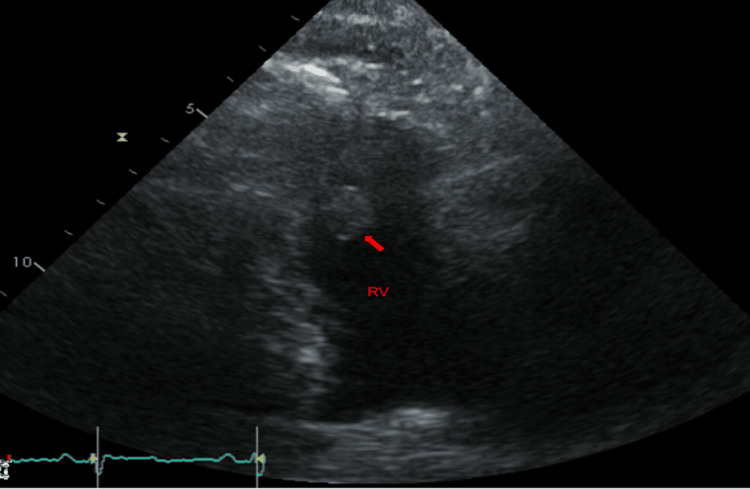
2D echocardiogram. Right ventricular (RV) cavity with a large mass.

**Figure 4:**
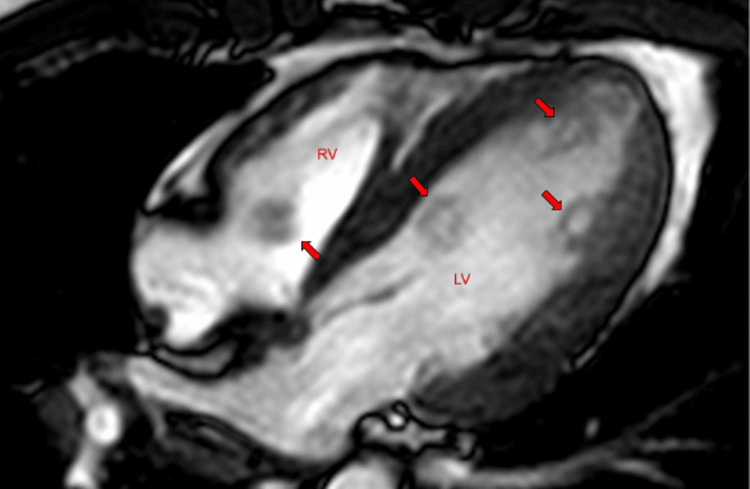
Cardiac MRI, left ventricular (LV) and right ventricular (RV) melanomas (arrows). Source: Ref. [8]

A 36-year-old Caucasian gentleman with a history of hypertension and an 18-pack-per-year smoking history presented with complaints of speaking gibberish and an inability to move his right hand, which lasted around 5-10 minutes and resolved spontaneously. His initial evaluation showed an elevated troponin and D-dimer. Physical examination was unremarkable except for axillary and cervical lymphadenopathy. He underwent a computed tomography (CT) scan of the chest to rule out pulmonary embolism, which showed a filling defect in the pulmonary trunk as well as the left ventricle. A head CT was negative for an acute infarct or hemorrhage. He had a transthoracic echocardiogram that showed multiple non-mobile masses attached to the left ventricular endocardium (Figures 1-2). Two masses were also seen in the right ventricle (Figure 3). The first one was attached to the base of the right ventricle just above the tricuspid valve. The second mass in the right ventricle was attached to the pulmonic valve, which protruded into the pulmonary artery during systole. He subsequently underwent cardiac magnetic resonance imaging (MRI) for further evaluation. The intracardiac masses demonstrated high intensity on T1 images, low intensity on T2 images, and a heterogeneous appearance on LGE imaging ( Figure 4). These characteristics were consistent with metastasis, particularly for malignant melanoma. Both his echocardiogram and cardiac MRI demonstrated normal biventricular systolic function. An MRI of the brain showed multifocal cerebral and a single cerebellar infarct of varying ages. His cerebrovascular accident (CVA) was thought to be cardioembolic in etiology. He had a CT scan of the chest, abdomen, and pelvis, which showed diffuse lymphadenopathy.

Oncology was consulted, and he underwent a cervical lymph node biopsy. Pathology was consistent with malignant melanoma, which was BRAF V600E mutation positive, PDL1 negative, and TMB intermediate. Detailed multiple marker panels showed PD-L1 28-8: negative, Pan-TRK: not expressed. NeoTYPE™ Melanoma FISH (fluorescence in situ hybridization) panel showed PTEN deletion: detected (1R1G, 32%, normal <26.6%), indicative of monosomy 10. Three clinically significant variants were detected, namely, BRAF V600E, CTNNB1 S45P, and TERT c.-124C>T, and PTEN deletion was positive. Microsatellite instability showed MSI-stable (MSS), PD-L1 28-8: negative, and tumor mutation burden: intermediate. He was treated with nivolumab and ipilimumab and tolerated them very well. Repeat imaging three months later showed stable cardiac metastasis without any evidence of disease progression. Currently, he is clinically doing well and regularly follows up with Oncology and Cardiology.

## Discussion

Melanoma is considered a masquerader of many diseases due to its potential to metastasize to many organs. These are aggressive tumors that can metastasize to any organ, but data from autopsy studies show common involvement in the heart (64%) [1]. Antemortem diagnosis of melanoma with cardiac metastasis is rare (<2%) as the symptoms are vague, and less than 16% of patients present with cardiac symptoms. Many factors contribute to the low rate of antemortem diagnosis, among them is the lack of distinct symptoms and difficulty identifying cardiac masses on traditional scans. Symptoms of melanoma with cardiac metastasis are usually silent, but obstruction, development of arrhythmia, congestive heart failure, or pericardial effusion can cause shortness of breath and palpitations [2]. Because these are symptoms of many cardiac and respiratory issues, melanoma with cardiac metastasis is often overlooked as a possible diagnosis for these patients. As a result, the cardiac MRI that is needed to rule out this diagnosis is often not ordered, and the diagnosis is missed altogether. The major hurdle in the treatment of melanoma with cardiac metastasis is the identification of the cardiac tumor. Identifying patients who are at risk for the disease will allow providers to determine when a biopsy is appropriate to fully rule out melanoma with cardiac metastasis.

Multimodality imaging is required for proper diagnosis. Echocardiography (transthoracic or transesophageal with 3D imaging) is usually the initial test, and cardiac MRI is essential in confirming metastatic disease [3]. Positron emission tomography (PET) and CT scan are also utilized. A biopsy is needed for definitive diagnosis. Among cardiac metastasis, involvement is noticed in the left ventricle (41.9%), right atrium (35.5%), and right ventricle (19.4%) [2]. The most common valve involved is the tricuspid valve. In symptomatic patients, surgical intervention can be considered depending on the tumor location and extent of invasion, with or without chemo/immunotherapy [4].

Gene mutation testing helps to choose the treatment. For example, BRAF gene mutations are found in 45% of melanoma cases, and targeted therapy led to a positive response in more than 60% of cases [5]. Serum lactate dehydrogenase (LDH) is one of the strongest independent prognostic factors in metastatic melanoma and has been incorporated into the evaluation of the disease [6]. After interventions, one study showed close to 60% of patients were disease-free without any evidence of recurrence. Recurrence occurred in 10% of patients, and 36% expired within 12 months [3]. Immune checkpoint inhibitors demonstrate meaningful efficacy, with close to 75% of patients having concordant responses with no overtly concerning safety signals [7]. In our case, we opted for immunotherapy without surgical intervention due to the presence of multiple tumors and the extent of the disease. Immune checkpoint inhibitors along with targeted therapy have greatly improved the outcomes in these cases. A multidisciplinary approach, more proactive diagnostic techniques, and close follow-up are essential in the management of this aggressive disease.

## Conclusions

Cardiac metastases with melanoma are common, and, given nonspecific clinical presentation, diagnosis usually is delayed. Prompt multimodality imaging and tissue diagnosis are crucial along with molecular testing, as noted in our case report. Treatment consists of chemotherapy and/or immunotherapy along with surgical intervention if appropriate.

## References

[1] Glancy DL, Roberts WC (1968). The heart in malignant melanoma: a study of 70 autopsy cases. Am J Cardiol.

[2] Balinski AM, Kerndt KC, Parry NP, Rehman R, Yeow R, Hayek S (2020). Metastatic melanoma of the heart: a systematic review. J Clin Oncol.

[3] Chiles C, Woodard PK, Gutierrez FR, Link KM (2001). Metastatic involvement of the heart and pericardium: CT and MR imaging. Radiographics.

[4] Hoffmeier A, Sindermann JR, Scheld HH, Martens S (2014). Cardiac tumors--diagnosis and surgical treatment. Dtsch Arztebl Int.

[5] Grimaldi AM, Simeone E, Festino L (2015). Novel mechanisms and therapeutic approaches in melanoma: targeting the MAPK pathway. Discov Med.

[6] Palmer SR, Erickson LA, Ichetovkin I, Knauer DJ, Markovic SN (2011). Circulating serologic and molecular biomarkers in malignant melanoma. Mayo Clin Proc.

[7] Nassar A, Alaiwi SA, Zarif TE (2023). Safety and efficacy of immune checkpoint inhibitors (ICIs) in patients (pts) with cardiac metastases (mets) from solid tumors. J Clin Oncol.

[8] Motwani M, Kidambi A, Herzog BA, Uddin A, Greenwood JP, Plein S (2013). MR imaging of cardiac tumors and masses: a review of methods and clinical applications. Radiology.

